# Presumed cytomegalovirus retinitis as the initial infectious manifestation of previously unrecognized Good syndrome in an HIV-negative patient: a case report

**DOI:** 10.3389/fmed.2026.1928863

**Published:** 2026-10-29

**Authors:** Chenyi Yang, Jiabei Xiong, Jiao Huang, Yongping Hu, Jianguang Zhong, Yuqin Wang, Jian Li

**Affiliations:** 1 The Fourth School of Clinical Medicine, Zhejiang Chinese Medical University, Hangzhou First People’s Hospital, Hangzhou, Zhejiang, China; 2 Department of Ophthalmology, Affiliated Hangzhou First People’s Hospital, School of Medicine, Westlake University, Hangzhou, Zhejiang, China; 3 Department of Rheumatology and Immunology, Affiliated Hangzhou First People’s Hospital, School of Medicine, Westlake University, Hangzhou, Zhejiang, China; 4 The Eye Hospital, Wenzhou Medical University, Wenzhou, Zhejiang, China

**Keywords:** case report, cytomegalovirus retinitis, good syndrome, immunodeficiency, thymoma

## Abstract

**Background:**

Cytomegalovirus (CMV) retinitis is a sight-threatening opportunistic infection that is uncommon in HIV-negative individuals without identifiable secondary cause of immunosuppression. Good syndrome (GS) is a rare adult-onset immunodeficiency associated with thymoma, and CMV retinitis as its initial infectious presentation is exceptionally rare.

**Case presentation:**

A 47-year-old HIV-negative woman presented with blurred vision in the right eye for 1 month. She had a history of thymectomy for type AB thymoma and left-eye pars plana vitrectomy (PPV) 2 years earlier. At presentation, best-corrected visual acuity (BCVA) was light perception (LP) in the right eye and no light perception (NLP) in the left eye. Fundus examination revealed extensive hemorrhagic necrotizing retinitis involving the posterior pole and macula. B-scan ultrasonography showed dense vitreous opacities and localized retinal detachment. Optical coherence tomography (OCT) showed retinal thickening, hyperreflective lesions, and disruption of the retinal architecture. The markedly elevated serum CMV DNA level, together with the characteristic fundus findings, supported a clinical diagnosis of presumed CMV retinitis. Intraocular polymerase chain reaction (PCR) confirmation was unavailable because the patient declined aqueous humor sampling. Immunologic evaluation demonstrated absence of peripheral CD19+ B cells, hypogammaglobulinemia, reduced CD4+ T-cell count, and an inverted CD4/CD8 ratio. In the setting of prior thymoma, these findings supported the diagnosis of GS. The patient was treated with intravenous immunoglobulin (IVIG) and intravenous ganciclovir. Serum CMV DNA levels declined substantially after treatment, but visual recovery was limited.

**Conclusions:**

Presumed CMV retinitis may be the initial infectious manifestation of previously unrecognized GS, with typical combined humoral and cellular immunodeficiency already evident at presentation. In HIV-negative patients with presumed CMV retinitis and no evident secondary immunosuppression, underlying immunodeficiency should be actively investigated. Particular attention should be paid to a history of thymoma, and early systemic immunologic evaluation should be considered to reduce diagnostic delay and irreversible visual loss.

## Introduction

Cytomegalovirus (CMV) retinitis is a sight-threatening opportunistic infection that typically occurs in patients with severe immunodeficiency, most commonly those with HIV infection. In HIV-negative patients without established secondary causes of immunosuppression, such as organ transplantation or long-term immunosuppressive therapy, CMV retinitis is uncommon and should prompt evaluation for an underlying immunodeficiency (1). Good syndrome (GS) is a rare adult-onset immunodeficiency associated with thymoma and characterized by combined defects in humoral and cellular immunity (2). Although CMV infection has been reported in patients with GS, CMV retinitis remains an uncommon infectious manifestation (3). To our knowledge, only one previous case has been reported in which CMV retinitis, in the absence of other systemic infections, was the presenting infectious manifestation leading to the diagnosis of GS (4, 5). Notably, our patient already exhibited a typical combined humoral and cellular immunodeficiency phenotype of GS at presentation.

## Case presentation

A 47-year-old woman was referred to the general ophthalmology ward of our hospital in October 2025 with blurred vision in the right eye for 1 month. Before referral, she had been diagnosed with retinitis in the right eye at another institution and treated with oral prednisone, without improvement. Her medical history was notable for thymectomy for a type AB thymoma 2 years earlier. In the same year, she underwent pars plana vitrectomy (PPV) in the left eye, the indication for which was unclear. She reported gradual visual loss in the left eye after surgery but could not recall its exact timing or progression. She had no history of HIV infection, organ transplantation, or long-term immunosuppressive therapy.

At presentation, best-corrected visual acuity (BCVA) was light perception (LP) in the right eye and no light perception (NLP) in the left eye. Intraocular pressure was 14.9 mmHg in the right eye and 13.1 mmHg in the left eye. Slit-lamp examination showed numerous keratic precipitates in both eyes, fixed dilated pupils without light reflexes, focal posterior synechiae, and lens opacities. In the right eye, dense vitreous opacity limited fundus visualization, but extensive confluent yellow-white necrotizing retinitis involving the posterior pole and macula was visible, accompanied by retinal hemorrhages, perivascular whitening, and vascular sheathing. In the left eye, severe vitreous haze completely obscured the fundus. B-scan ultrasonography of the right eye showed vitreous opacity, localized retinal detachment, and proliferative membranous changes (Figure 1). Optical coherence tomography (OCT) showed retinal thickening, hyperreflective lesions, and disruption of the retinal architecture.

**Figure 1:**
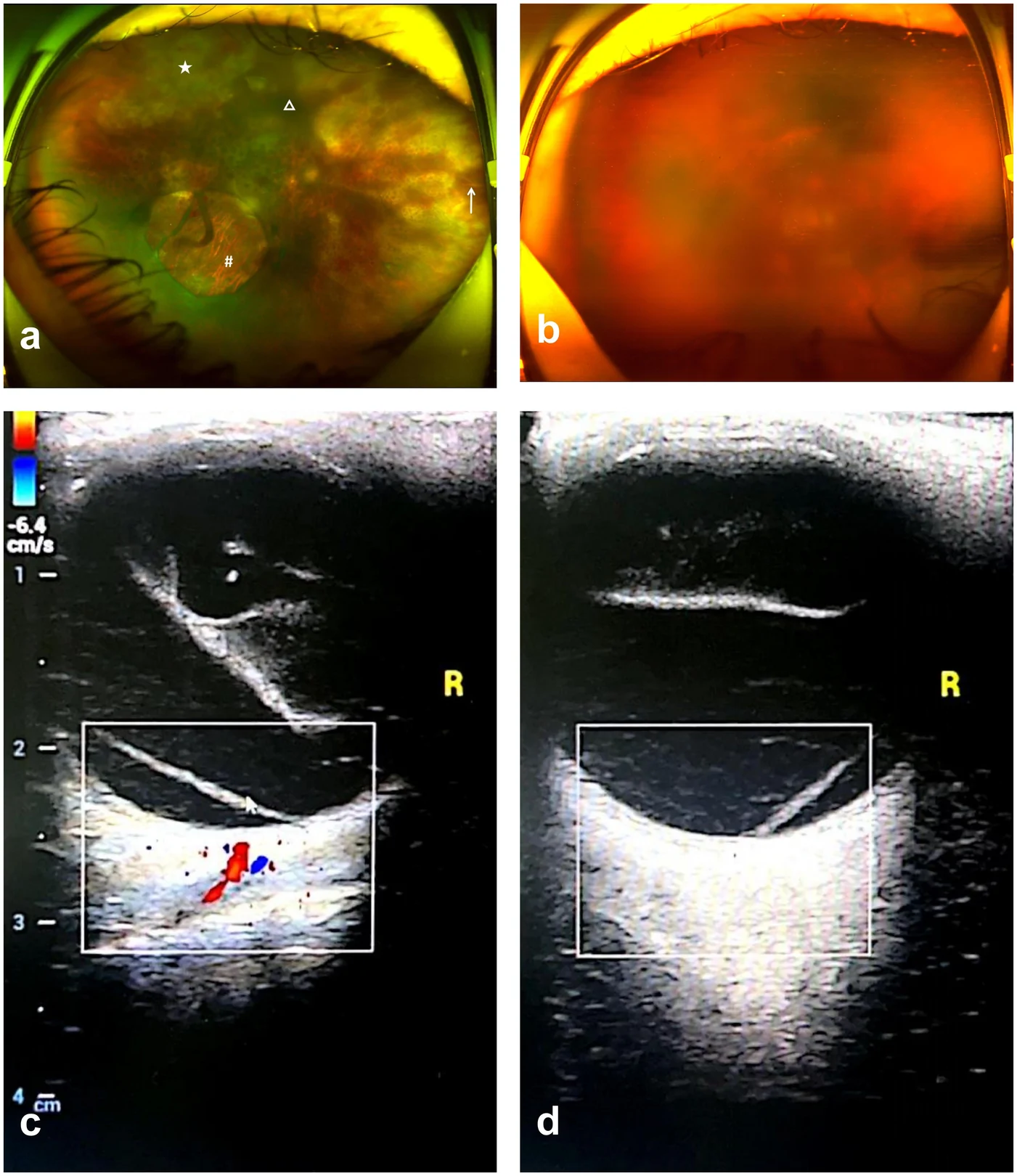
Fundus and ocular ultrasound findings at presentation. **(a)** Right eye: Dense vitreous opacity markedly limited fundus visualization. The posterior pole and macular region showed extensive confluent yellow-white necrotizing retinitis with scattered intraretinal hemorrhages, creating a characteristic “cottage cheese and ketchup” or “pizza pie” appearance. Perivascular whitening, vascular sheathing, and focal vascular attenuation were also present. Hash signs indicate choroidal atrophy; white arrows indicate perivascular and vascular changes; triangles indicate retinal hemorrhages; and asterisks indicate necrotizing retinitis. **(b)** Left eye: Dense vitreous opacity precluded fundus visualization. **(c,d)** Right eye B-scan ultrasonography: Dense vitreous echoes were present, together with apparently longstanding localized retinal detachment, visible as a hyperreflective membranous structure. Additional membranous echoes suggested proliferative vitreoretinal changes.

On October 16, 2025, at admission to the general ophthalmology ward, a provisional diagnosis of necrotizing retinitis of uncertain etiology was made for the right eye. Because of prominent intraocular inflammation, the patient received mydriatics, topical ganciclovir gel, and an empiric course of intravenous methylprednisolone at 250 mg/day for 5 days. As the ocular condition did not improve, she was transferred to our uveitis subspecialty ward on October 21. Based on the characteristic fundus findings, our uveitis subspecialty team strongly suspected CMV retinitis and considered the possibility of an underlying immunodeficiency. Intravenous methylprednisolone was immediately discontinued, and intravenous ganciclovir was initiated on October 21 at 300 mg twice daily, based on her normal renal function and body weight (62.5 kg), concurrently with serum CMV DNA testing and immunological evaluation.

Additional infectious and autoimmune investigations were performed to evaluate alternative causes of necrotizing retinitis, including HIV antibody, Epstein–Barr virus DNA, Treponema pallidum antibody, T-SPOT.TB, TORCH testing, and autoimmune antibody tests. HSV IgG was positive, and HSV IgM was negative. Other TORCH tests, including Toxoplasma gondii IgG and IgM, were negative. Other systemic investigations were unremarkable. The serum CMV DNA result became available on October 22 and showed a markedly elevated level of 9.29 × 10^5^ IU/mL. Although aqueous humor PCR was recommended for intraocular microbiological confirmation, the patient declined the procedure. Based on the characteristic fundus findings and markedly elevated serum CMV DNA level, presumed CMV retinitis was clinically diagnosed, and intravenous ganciclovir was continued.

Initial laboratory testing showed lymphopenia. Further immunologic evaluation (Table 1) demonstrated a complete absence of peripheral CD19+ B cells and hypogammaglobulinemia, with reduced serum IgG, IgA, and IgM levels. The CD4+ T-cell count was also decreased (102.81 cells/*μ*L), with an inverted CD4/CD8 ratio (0.39), consistent with combined humoral and cellular immune deficiency. In the context of her history of thymoma, these findings supported the diagnosis of GS. Intravenous immunoglobulin (IVIG) was initiated on October 23 at 20 g/day for 2 days, with peribulbar triamcinolone acetonide (20 mg every 2 weeks) added as adjunctive treatment.

**Table 1:** Immunological findings at presentation.

Parameter	Value	Unit	Reference range
Total lymphocyte count	434.78	cells/μL	1200–3400
CD3^+^ T cells	404.58	cells/μL	690–1760
CD4^+^ T cells	102.81	cells/μL	410–884
CD8^+^ T cells (%)	62.26	%	15–44
CD4/CD8 ratio	0.39	-	0.71–2.87
CD19^+^ B cells	0	cells/μL	90–323
CD19^+^ B cells (%)	0	%	5–18
NK cells (CD16^+^/CD56^+^)	27.43	cells/μL	90–536
IgG	3.47	g/L	8.6–17.4
IgA	0.19	g/L	1.0–4.2
IgM	0.39	g/L	0.5–2.8

Reference ranges are based on the laboratory standards of our institution.

Given that the lungs and gastrointestinal tract are commonly involved sites of CMV infection in patients with GS (4), targeted evaluations of these organ systems were performed. Chest CT showed no evidence of pulmonary involvement, and CMV PCR of bronchoalveolar lavage fluid was negative. Gastrointestinal endoscopy with mucosal biopsy showed no evidence of CMV infection. In this patient, presumed CMV retinitis was the initial infectious manifestation of previously unrecognized GS. No evidence of CMV involvement in other commonly affected organ systems was identified.

According to current international consensus and guidelines (8), in immunocompromised patients with a high clinical suspicion of CMV retinitis, particularly when the lesions involve the central posterior retina, a more aggressive antiviral treatment strategy may be considered, including prompt initiation of systemic anti-CMV therapy with adjunctive intravitreal ganciclovir. Given that this patient met the above indications for intravitreal therapy, intravitreal ganciclovir was recommended to achieve a higher intraocular antiviral drug concentration and more rapid local viral suppression. Given the proliferative membrane, PPV was also recommended to potentially improve the ocular prognosis. Despite detailed counseling regarding the risks of continued disease progression and poor visual prognosis, the patient declined both interventions.

On October 25, serum CMV DNA had declined from 9.29 × 10^5^ to 2.48 × 10^5^ IU/mL and continued to decrease thereafter (Figure 2). The retinal lesions appeared consolidated with residual atrophic changes during intravenous anti-CMV therapy, despite the marked decline in serum CMV DNA (Figure 3). Vision in the right eye subsequently deteriorated to NLP at the final follow-up. On October 25, the patient was transferred to the Department of Rheumatology and Immunology for further treatment. Intravenous ganciclovir was administered at 300 mg twice daily from October 21 to October 31. On November 1, she requested discharge and was discharged on oral ganciclovir (0.5 g three times daily) for maintenance antiviral therapy.

**Figure 2:**
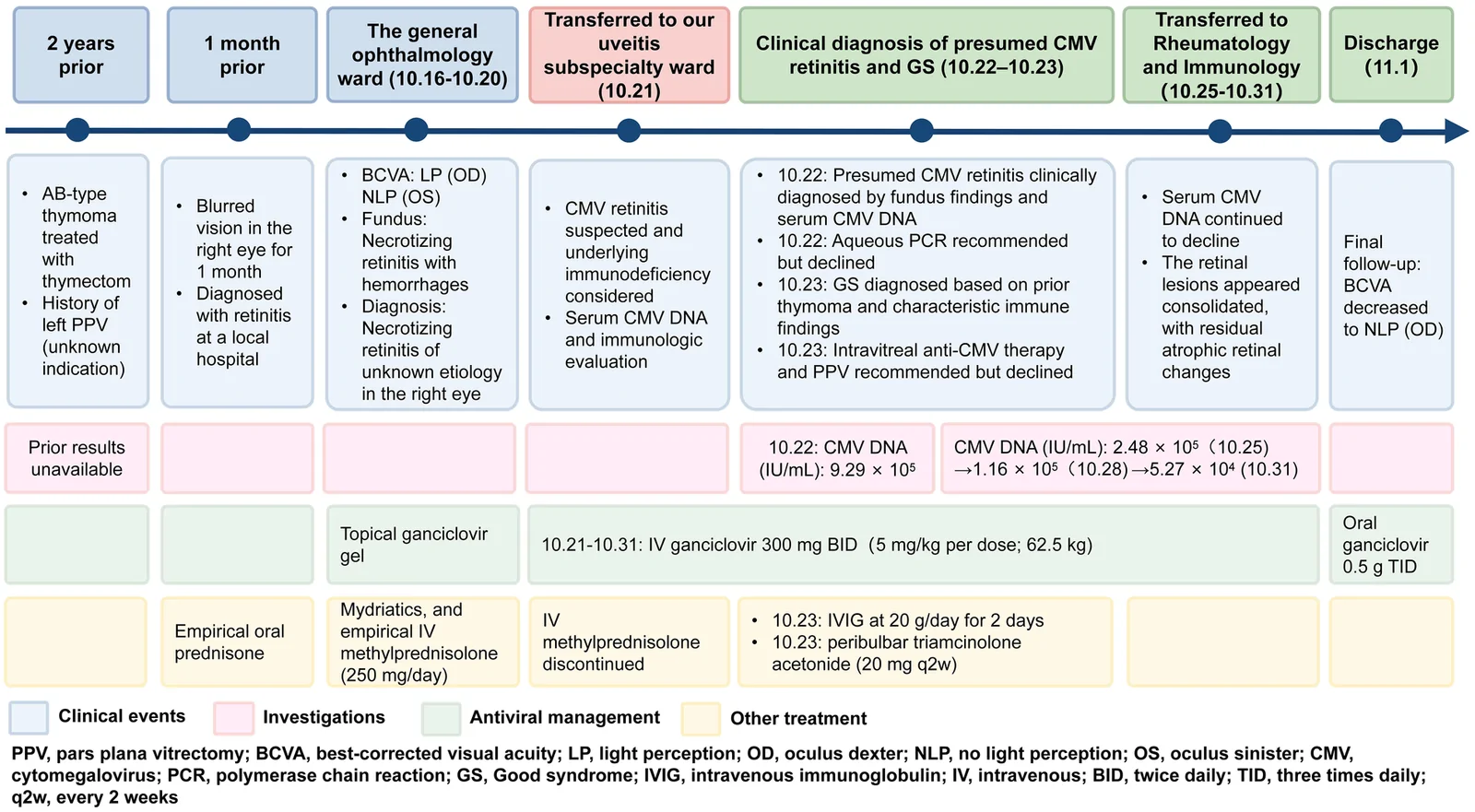
Timeline of the patient's clinical course.

**Figure 3:**
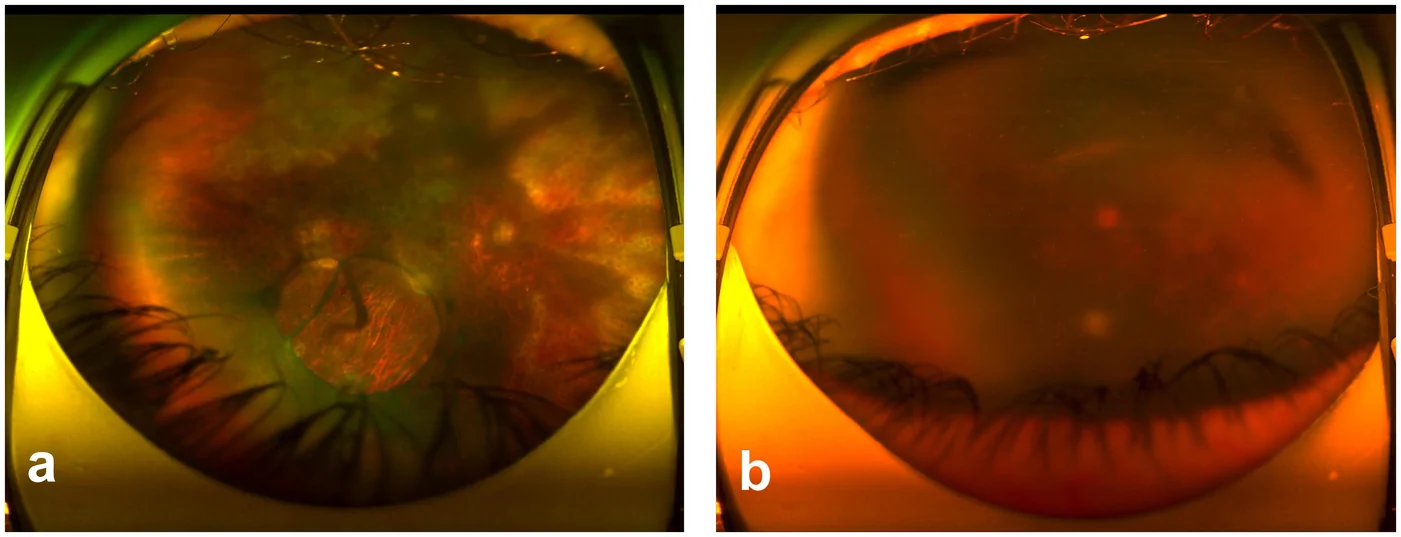
Fundus photographs after treatment. **(a)** Right eye: The retinal lesions appeared consolidated, with residual atrophic retinal changes. Retinal hemorrhages and vascular abnormalities remained evident in the posterior pole and macula. **(b)** Left eye: Dense vitreous opacity continued to preclude adequate fundus visualization.

## Discussion

GS is a rare immunodeficiency associated with thymoma, characterized by combined humoral and cellular immune defects, predisposing patients to opportunistic infections including CMV infection (2, 6). Although CMV infection has been well documented in GS, CMV retinitis remains an exceptionally rare ocular manifestation (3). A recent review identified fewer than 20 reported cases worldwide, most of which were unilateral and associated with poor visual outcomes (4, 7). CMV retinitis as the presenting feature of GS without other systemic infections is extremely rare, with only a very limited number of cases having been reported (5). Unlike the previously reported case, in which peripheral T-cell counts remained within the normal range, our patient already exhibited a typical combined humoral and cellular immunodeficiency phenotype at presentation, including complete absence of peripheral B cells, hypogammaglobulinemia, CD4+ T-cell depletion, and an inverted CD4/CD8 ratio. These findings suggest that clinically significant combined immune dysfunction may already be established when presumed CMV retinitis is the initial infectious manifestation of GS.

According to the current consensus and guidelines (8), the clinical diagnosis of CMV retinitis is based on patient susceptibility due to compromised immune function and characteristic fundus manifestations. In this case, both cellular and humoral immunity were impaired, and the fundus showed characteristic CMV retinitis lesions involving the posterior pole and macula, with hemorrhagic and necrotizing changes. Although intraocular PCR could not be performed because the patient declined aqueous humor sampling, the markedly elevated serum CMV DNA level and its subsequent decline following antiviral therapy provided laboratory support for presumed CMV retinitis. Furthermore, the consolidation of active necrotizing lesions into stable atrophic scars following antiviral intervention is a characteristic retinal response, which further corroborates our presumed diagnosis of CMV retinitis.

Other causes of necrotizing retinitis were also considered in the differential diagnosis, including acute retinal necrosis, progressive outer retinal necrosis, and ocular toxoplasmosis. Acute retinal necrosis typically begins in the peripheral retina and progresses circumferentially. In contrast, this patient presented with extensive confluent necrotizing retinitis involving the posterior pole and macula, without serological evidence of recent HSV infection. Progressive outer retinal necrosis is characterized by rapidly progressive outer retinal involvement and typically lacks prominent retinal vasculitis, whereas this patient had prominent retinal vascular inflammation and a relatively prolonged clinical course. Ocular toxoplasmosis more commonly presents as focal necrotizing retinal lesions, whereas this patient had extensive necrotizing retinitis, and both Toxoplasma gondii IgG and IgM were negative. Overall, these clinical and laboratory findings were less consistent with the major alternative diagnoses.

In an HIV-negative patient with presumed CMV retinitis and no history of organ transplantation or long-term immunosuppressive therapy, evaluation for an underlying immunodeficiency is warranted. In this case, the combination of hypogammaglobulinemia and absent peripheral B cells, in the setting of thymoma, supported a diagnosis of GS. However, although immunological investigations had been performed at the time of thymoma diagnosis, the corresponding results were no longer available, and the patient reported that she had not undergone follow-up after thymectomy. Therefore, it remains unclear whether the immunodeficiency was already present but unrecognized at the time of thymoma diagnosis or developed subsequently.

From a management perspective, thymectomy does not correct the underlying immune defect in GS (9), and infectious complications may occur both before and years after tumor resection (1, 4). The development of presumed CMV retinitis 2 years after thymectomy in this patient further supports the persistence of immunodeficiency despite tumor removal. Accordingly, long-term immunologic follow-up, immunoglobulin replacement, and targeted anti-infective therapy are recommended.

IVIG provides passive humoral immune support and is a key component of infection management in GS (9, 10). In the present case, intravenous ganciclovir combined with IVIG led to a marked decline in serum CMV DNA, but no visual recovery was achieved, and vision in the right eye ultimately deteriorated to NLP. Several factors likely contributed to this poor outcome. First, at presentation, the right eye already showed extensive confluent necrotizing retinitis involving the macula, together with localized retinal detachment and proliferative membranous changes, suggesting that irreversible retinal damage had already occurred (11). Second, corticosteroid therapy was initiated before the infectious etiology was clarified, which may have delayed definitive antiviral treatment. Third, in HIV-negative patients, partially preserved anti-CMV immunity may lead to a more pronounced inflammatory response, resulting in diagnostic difficulty and delayed treatment, thereby worsening visual prognosis (1).

Retinal detachment represents a severe, late-stage sight-threatening complication of CMV retinitis, indicating extensive retinal necrosis and poor visual prognosis. Its occurrence necessitates individualized antiviral strategies and aggressive surgical intervention, as well as prolonged fundus surveillance owing to the high risk of persistent or delayed retinal breaks and redetachment. In the present case, presumed CMV retinitis was still active when retinal detachment was detected. Extensive retinal necrosis, together with a localized retinal detachment that appeared longstanding, suggested that substantial retinal damage was already irreversible, and meaningful visual recovery was therefore unlikely even if anatomical reattachment were achieved. Given the limited potential for visual recovery and the patient's financial constraints, retinal detachment repair was not pursued.

Bilateral CMV retinitis is well recognized in HIV infection and other secondary immunosuppressive states but has rarely been reported in GS (7). The patient had previously undergone PPV in the left eye for an undocumented indication, and the eye had NLP at presentation. Because detailed ophthalmic records were unavailable, the underlying etiology could not be determined. Although bilateral CMV retinitis could not be confirmed, the possibility of bilateral ocular involvement cannot be excluded in the setting of profound combined immunodeficiency. Earlier recognition of the underlying immunodeficiency may facilitate timely diagnosis and treatment, potentially reducing the risk of irreversible visual loss.

Aqueous humor PCR was recommended to obtain intraocular microbiologic evidence (8), but the patient declined the procedure, precluding microbiologic confirmation of intraocular CMV infection. Delayed diagnosis and initial management may have contributed to the poor outcome, as antiviral treatment could not reverse established retinal damage. A further limitation is the short follow-up period, which precluded assessment of long-term immune status and recurrence risk.

## Conclusion

Presumed CMV retinitis may be the presenting infectious manifestation of previously unrecognized GS, even in the absence of other systemic infections. In this patient, marked combined humoral and cellular immune defects were already evident at presentation. These findings support early immunologic evaluation in HIV-negative patients with presumed CMV retinitis, particularly those with a history of thymoma.

## Data Availability

The original contributions presented in the study are included in the article/Supplementary Material, further inquiries can be directed to the corresponding authors.
